# Temporal Trends of Emergency Department Visits of Patients with Atrial Fibrillation: A Nationwide Population-Based Study

**DOI:** 10.3390/jcm9051485

**Published:** 2020-05-14

**Authors:** So-Ryoung Lee, Eue-Keun Choi, Seo-Young Lee, Euijae Lee, Kyung-Do Han, Myung-Jin Cha, Woon Yong Kwon, Sang Do Shin, Seil Oh, Gregory Y. H. Lip

**Affiliations:** 1 Department of Internal Medicine, Seoul National University Hospital, Seoul 03080, Korea; minerva1368@gmail.com (S.-R.L.); sizuku27@gmail.com (S.-Y.L.); euijae14@gmail.com (E.L.); cmj.with.love@gmail.com (M.-J.C.); seil@snu.ac.kr (S.O.); 2 Department of Internal Medicine, Seoul National University College of Medicine, Seoul 03080, Korea; Gregory.Lip@liverpool.ac.uk; 3 Statistics and Actuarial Science, Soongsil University, Seoul 03080, Korea; hkd917@naver.com; 4 Department of Emergency Medicine, Seoul National University Hospital, Seoul 03080, Korea; kwy711@snu.ac.kr (W.Y.K.); sdshin@snu.ac.kr (S.D.S.); 5 Liverpool Centre for Cardiovascular Science, University of Liverpool and Liverpool Chest & Heart Hospital, Liverpool L143PE, UK; 6 Department of Clinical Medicine, Aalborg University, 9000 Aalborg, Denmark

**Keywords:** atrial fibrillation, emergency department, stroke, oral anticoagulant

## Abstract

We aimed to describe temporal trends in emergency department (ED) visits of patients with atrial fibrillation (AF) over 12 years. A repeated cross-sectional analysis of ED visits in AF patients using the Korean nationwide claims database between 2006 and 2017 were conducted. We identified AF patients who had ≥1 ED visits. The incidence of ED visits among total AF population, cause of ED visit, and clinical outcomes were evaluated. During 12 years, the annual numbers of AF patients who attended ED at least once a year continuously increased (40,425 to 99,763). However, the annual incidence of ED visits of AF patients was stationary at about 30% because the number of total AF patients also increased during the same period. The most common cause of ED visits was cerebral infarction. Although patients had a higher risk profile over time, the 30-day and 90-day mortality after ED visit decreased over time. ED visits due to ischemic stroke, intracranial hemorrhage, and myocardial infarction decreased, whereas ED visits due to AF, gastrointestinal bleeding, and other major bleeding slightly increased among total AF population over 12 years. A substantial proportion of AF patients attended ED every year, and the annual numbers of AF patients who visited the ED significantly increased over 12 years. Optimized management approaches in a holistic and integrated manner should be provided to reduce ED visits of AF patients.

## 1. Introduction

Atrial fibrillation (AF) is the most common cardiac arrhythmia, and its prevalence is increased by the aging population and aging-related comorbidities [1,2,3,4]. AF is associated with an increased risk of mortality and morbidity due to heart failure, dementia, and ischemic stroke compared to patients without AF [4,5,6]. Comorbid conditions complicated by AF caused hospitalization, and consequently, the healthcare burden associated with AF is growing [7,8,9]. Previous studies, mainly from the United States or Europe, have reported an increase in hospitalizations and total cost for AF care in recent decades [7,8]. In Korea, hospitalizations for AF have increased by 4.2-fold, and the total cost of care increased by about 5.7-fold over the past 10 years [9].

While the healthcare burden of AF is mainly related to hospitalization [10], an emergency department (ED) visit is an indicator that reflects poorly controlled AF symptoms or complications of AF and is associated with worsened quality of life. A substantial number of patients who visit an ED are subsequently hospitalized [11,12]. Despite the clinical impact of ED visits in patients with AF, most epidemiologic studies have been based on Western populations, with limited data in Asian populations [11,12,13].

Nationwide data focusing on temporal trends, characteristics, and clinical consequence of ED visit of patients with AF are essential for optimal healthcare planning. Therefore, we evaluated the temporal trends in ED visits of patients with AF, patients’ characteristics, causes of ED visits, and clinical outcomes using a nationwide insurance claims database in Korea.

## 2. Materials and Methods

### 2.1. Data Source and Study Population

This study used the national health claims database established by the Korean National Health Insurance Service (NHIS) [14]. The NHIS of Korea contains the demographic and medical claim information of approximately the entire 50 million Korean population. Korea has a single-payer universal and compulsory health coverage system, which covers the entire Korean population. It contains patients’ demographics, including date of birth and death, diagnoses, examinations, procedures, surgeries, and prescriptions for inpatient and outpatient services. Diagnoses were coded using the International Classification of Disease, Tenth Revision, Clinical Modification (ICD-10-CM) codes. This study was exempted from review by the Seoul National University Hospital Institutional Review Board (1909-089-1065).

From January 2006 to December 2017, we conducted repeated cross-sectional retrospective observational cohorts of adult patients with non-valvular AF in each index year [3,12]. AF was defined as diagnostic codes I48.0–I48.4, I48.9, and patients with mitral stenosis or preexisting prosthetic heart valves were excluded (Table S1) [3,9,15]. When defining AF patients who visited ED, firstly, we identified total patients with non-valvular AF in the entire Korean adult population. Then, among these populations, we identified patients with any cause of ED visit at least once in a year. The type of AF has limited accuracy in the claims database because this information did not affect the reimbursement. Also, atrial flutter often coexists with or precedes AF [16], and the stroke risk and the goals for the management of atrial flutter are not much different from that in AF [17,18]. Therefore, we included all AF types (paroxysmal, persistent, or permanent) and atrial flutter as an operational definition of AF in this study. Among the whole AF population, we identified patients who visited ED ≥1 during the index year.

### 2.2. Covariates

We collected patients’ baseline demographics and comorbidities during 1 year before the index year. Detailed definitions of comorbidities are presented in Table S1 [3,9,19]. Comorbidities included hypertension, diabetes mellitus, heart failure, stroke/transient ischemic attack (TIA)/thromboembolism (TE), myocardial infarction (MI), peripheral artery disease (PAD), chronic obstructive pulmonary disease (COPD), chronic kidney disease (CKD), and end-stage renal disease with renal replacement therapy. The CHA_2_DS_2_-VASc scores were calculated by assigning 1 point for heart failure, hypertension, diabetes mellitus, vascular disease (prior MI or PAD), age ≥65 years, and female sex and assigning 2 points for previous stroke/TIA/TE and age ≥75 years [20]. We also evaluated patients’ prescriptions during the index year. For patients with ED visits, the number of ED visits in the index year was described.

### 2.3. Clinical Outcomes after ED Visits

Subsequent all-cause hospitalization after ED visits were identified, and the proportion of subsequent hospitalization after ED visits among total ED visits was calculated. After identifying the index ED visits (the first ED visits during the index year), 30-day and 90-day all-cause mortality were assessed among all patients with ED visits in the index year.

### 2.4. Causes of ED Visits

The cause of ED visit was defined as the primary diagnosis of the index ED visit. To evaluate the temporal trends of the common causes of ED visits, we assessed the ten most common primary diagnosis codes among total ED visits each year.

### 2.5. Definition of ED Visits from AF-Related Complications

ED visits from ischemic stroke, AF, heart failure, MI, intracranial hemorrhage (ICH), gastrointestinal (GI) bleeding, or other major bleeding as a primary diagnosis of the index ED visits were defined as ED visits due to AF-related complications [19,21]. Detailed definitions of AF-related complications are shown in Table S1. To evaluate the temporal trends of ED visits for AF-related complications, the incidence of ED visit for AF-related complications was assessed by the number of patients with ED visits for AF-related complications per 100 patients with AF in each index year, presented as a percentage.

### 2.6. Statistical Analysis

Data management and statistical analyses were performed using SAS 9.3 (SAS Institute Inc., Cary, NC, USA). Continuous variables are presented as mean ± standard deviation, and categorical variables are presented as a percentage. The incidence of ED visits was calculated using the number of patients with ED visits at least once in the index year divided by the total AF population in each index year, presented as a percentage. For categorical variables, the Cochran–Mantel–Haenszel test was performed to analyze temporal trends. Based on the central limit theorem considering a large sample size of our cohort, we used a parametric method to evaluate p-for-trend. Thus, we performed a generalized linear model for continuous variables. For all statistical analyses, statistical significance was defined as a *p*-value of < 0.05.

## 3. Results

### 3.1. Summary of Total AF Population during the Study Period

**Figure 1:**
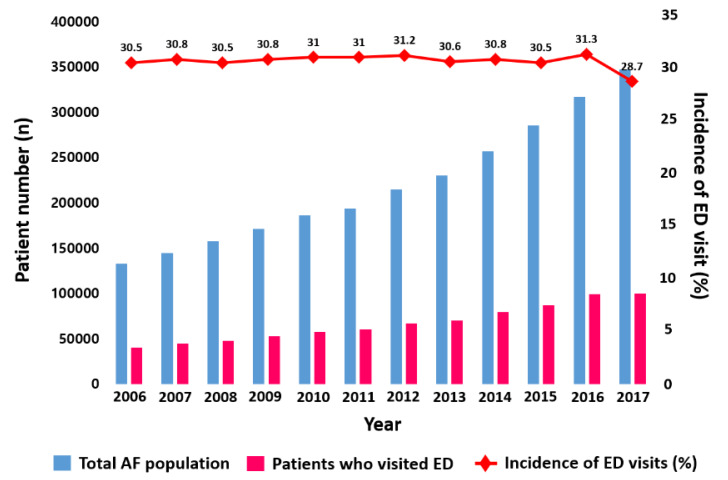
Number of total AF population, patients who visited ED from 2006 to 2017, and incidence of emergency department visits in Korean AF population. Abbreviations: AF, atrial fibrillation; ED, emergency department.

Extended from our previous report of AF epidemiology in the Korean population (from 2008 to 2015) [3], the number of patients with AF continuously increased from 2006 to 2017 (132,548 to 347,709) (Figure 1 and Table S2). Consistent with our previous findings, the mean age (66 to 71 years) and mean CHA_2_DS_2_-VASc score (2.9 to 3.4) of patients with AF increased, as the crude prevalence of comorbidities including hypertension, diabetes, heart failure, stroke/TIA/TE, PAD, and CKD were increased. Patients with prior MI decreased over time. Patients treated with oral anticoagulants (OACs) significantly increased from 29.1% to 55.0% over 12 years, associated with an increase of non-vitamin K antagonist oral anticoagulants (NOACs) prescriptions since 2015.

### 3.2. Incidence of ED Visits Among Total AF Population and Patients’ Baseline Characteristics

**Figure 2:**
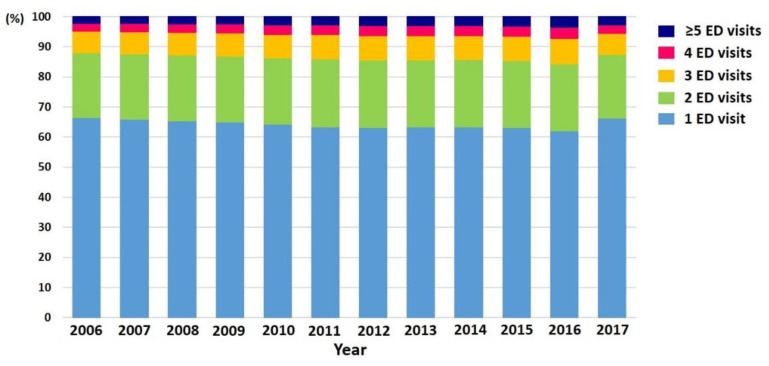
Patient distribution by the number of ED visits each year. Abbreviation: ED, emergency department.

With the overall AF population growing, the annual number of AF patients with ED visits significantly increased during the study period (40,425 in 2006 to 99,762 in 2017; 247% increase, *p*-for-trend < 0.001) (Figure 1). During a 12-year period, approximately 30% of all AF patients visited the ED at least once in a year (Figure 1 and Table S3). Among patients with ED visits, about 35% of patients visited the ED more than two times in a year (Figure 2 and Table 1). The mean number of ED visits was 1.6 ± 1.3 in 2017.

The mean age of patients with ED visits increased from 2006 to 2017 (68 to 73 years) and the prevalence of comorbidities including hypertension, diabetes, heart failure, previous stroke/TIA/TE, PAD, CKD increased, thus the mean CHA_2_DS_2_-VASc scores became higher from 2006 to 2017 (3.1 to 3.8) (Table 1). The rate of patients receiving OAC therapy became higher over time (29.5% to 59.5%). In 2006, warfarin was the only available OAC therapy, while more recently, NOACs have become the more prevalent OAC type (8.2% treated with warfarin and 51.3% treated with NOACs among patients with ED visits in 2017). The pattern of antiarrhythmic agent use has changed over time. The use of class I and III antiarrhythmic agents continuously increased from 2006 to 2017 (both *p*-for-trend <0.001). However, digoxin use has decreased over time (44.4% in 2006 and 25.8% in 2017, *p*-for-trend <0.001).

In 2017, compared to patients who did not visit ED, patients with ED visits generally were older, more likely to be women, showed a higher prevalence of comorbidities, and had a higher CHA_2_DS_2_-VASc score (Table S4). The rate of OAC use was higher in AF patients with ED visits than in those who did not attend ED (Table S4).

### 3.3. Clinical Outcomes after ED Visits

Among all ED visits of patients with AF, 65% to 75% of ED visits resulted in subsequent hospitalizations (Table S3). The hospitalization rates gradually declined from 73.3% in 2006 to 65.0% in 2015 but increased after 2015 (from 65.0% in 2015 to 75.1% in 2017). Overall, the annual volume of hospitalizations from ED visits in AF patients constantly increased during the study period by 259% (*p*-for-trend < 0.001).

**Figure 3:**
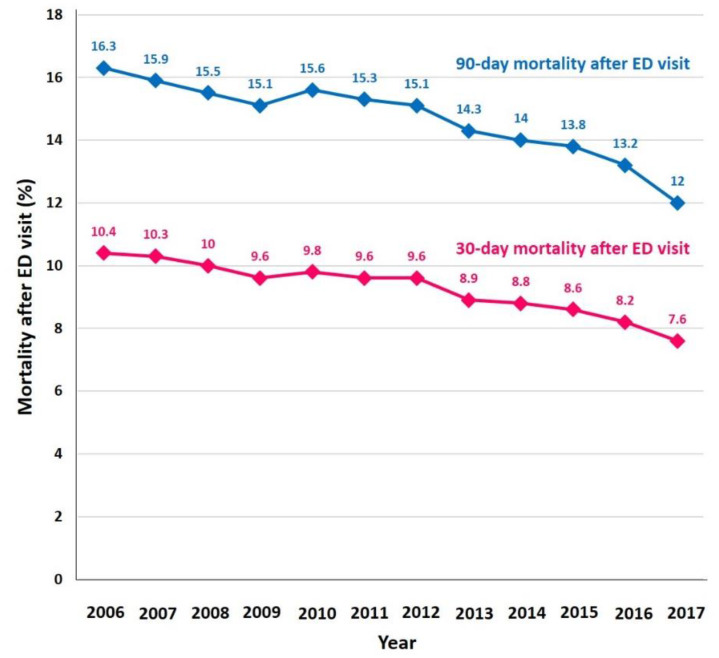
30 day and 90 day mortality after ED visit. Abbreviations: AF, atrial fibrillation; ED, emergency department.

A substantial proportion of AF patients with ED visits died during the 30-day and 90-day follow-up (Figure 3 and Table S3). In 2006, 30-day mortality of patients who visited the ED was 10.4%, and 90-day mortality was 16.3%. Temporal trends of 30-and 90-day mortality are shown in Figure 3. Although patients had a higher risk profile over time, 30-day mortality after the index ED visit showed a decreasing trend from 10.4% to 7.6% (*p*-for-trend < 0.001) during the study period. Similarly, 90-day mortality showed a decreasing trend from 16.3% to 12% during the study period.

### 3.4. Causes of ED Visits

**Figure 4:**
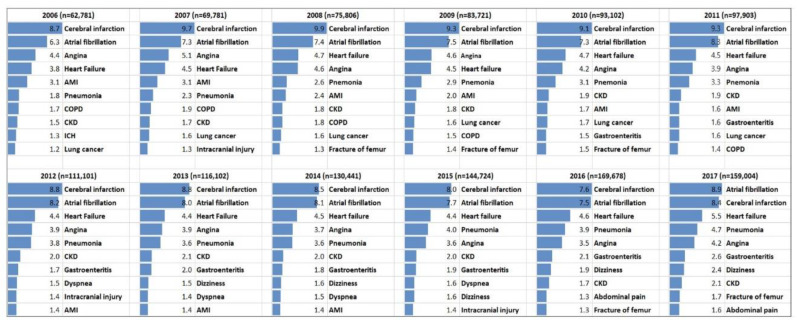
Common causes of ED visits in patients with AF. Abbreviations: AF, atrial fibrillation; AMI, acute myocardial infarction; CKD, chronic kidney disease; COPD, chronic obstructive pulmonary disease; ICH, intracranial hemorrhage.

In 2006, cerebral infarction (ICD-10-CM code, I63), AF (I48), angina (I20), heart failure (I50), and acute MI (I21) were the five most common causes of ED visits in AF patients (Figure 4). These five primary diagnoses were consistently ranked highly as common causes of ED visits. ICH (I62); non-cardiovascular causes including pneumonia (J18), COPD (J44), CKD (N18), lung cancer (C34), and gastroenteritis (A09); non-specified symptoms such as dyspnea (R06), dizziness (R42), and abdominal pain (R10); and trauma-related diagnoses including intracranial injury (S06) and fracture of femur (S72) were among the ten most common causes of ED visits in AF patients. From 2006 to 2016, cerebral infarction was the most common cause of ED visits, while AF, cerebral infarction, heart failure, pneumonia, and angina were the five most common causes in 2017, respectively.

### 3.5. Incidence of ED Visits from AF-Related Complications

**Figure 5:**
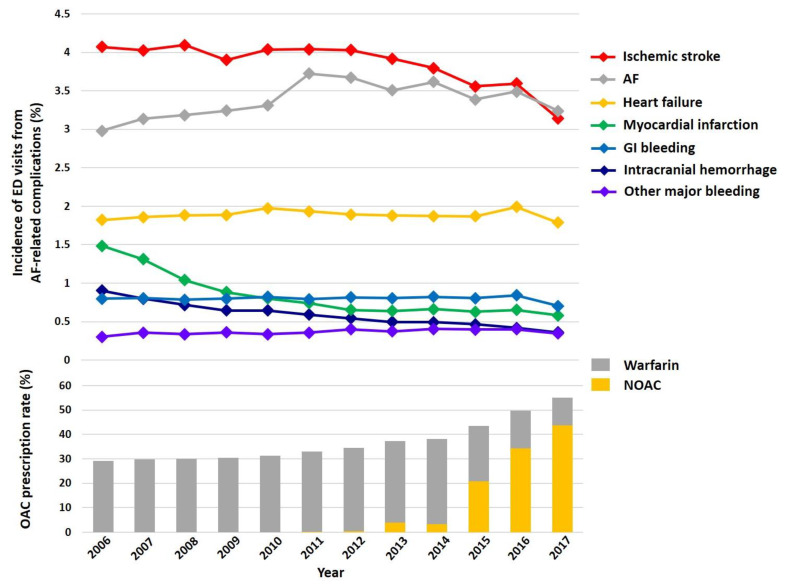
Temporal trends of incidence of ED visits from AF-related complications and OAC prescription rate among total AF population from 2006 to 2017. Abbreviations: AF, atrial fibrillation; ED, emergency department; GI, gastrointestinal; ICH, intracranial hemorrhage; NOAC, non-vitamin K antagonist oral anticoagulant; MI, myocardial infarction; OAC, oral anticoagulants.

Figure 5 shows the temporal trends of the incidence of ED visits from AF-related complications from 2006 to 2017. Incidence of ED visits due to ischemic stroke, ICH, and MI were significantly decreased during the study period (*p*-for-trend for ischemic stroke < 0.001; for ICH < 0.001; and for MI < 0.001). Incidence of ED visits due to AF showed fluctuations over time and an overall increase from 2.98% in 2006 to 3.24% in 2017 (*p*-for-trend < 0.001). The number of patients with ED visits from AF-related complications is described in Table S5. ED visits due to heart failure remained stable (*p*-for-trend = 0.701). GI bleeding and other major bleeding events slightly increased until 2016 but declined in 2017 (*p*-for-trend for GI bleeding = 0.058; and other major bleeding < 0.001).

## 4. Discussion

To the best of our knowledge, this is the first contemporary nationwide study of the temporal trends of ED visits of Asian patients with AF. The main findings of this study are as follows: (1) the annual number of AF patients with ED visits significantly increased during the study period, and approximately 30% of all AF patients visited the ED at least once in a year; (2) 35% of patients visited the ED more than 2 times in a year, and 65% to 75% of ED visits resulted in subsequent hospitalizations; (3) a substantial proportion of AF patients with ED visits died during the 30 -day and 90 day follow-up; (4) although patients had a higher risk profile over time, 30 and 90 day mortality after the index ED visit showed decreasing trends during the study period; and (5) incidences of ED visits due to ischemic stroke, ICH, and MI have decreased.

Globally, the number of patients with AF is increasing, with a consequent increase in healthcare burden associated with AF [1,2,3,4,7,8,9]. Although the prevalence of AF in the Asian population is generally lower (ranged from 0.7% to 1.6%) compared to Western populations, Asian data show a rapid increase of AF prevalence in recent decades with the aging population [1,3,4,22,23]. The natural course of AF requires lifelong management, including stroke prevention, rhythm and rate control, and management of comorbidities [24,25,26]. AF is also associated with increased risks of several adverse outcomes. In a recent meta-analysis, including 587,867 AF patients who were mainly Caucasians, AF was associated with increased risks of ischemic stroke by 2.3-fold, heart failure by 5-fold, ischemic heart disease by 1.6-fold, and all-cause death by 1.5-fold [27]. In a large-scale nationwide cohort study from an Asian population, AF was also associated with increased relative risks for adverse events, being generally higher for ischemic stroke (3.3 versus 2.3) and all-cause death (2.6 versus 1.5); lower for heart failure (3.3 versus 5.0); and similar for ischemic heart disease (1.6 versus 1.6) compared with adverse events in the Caucasian population [4,27].

Although AF is a chronic condition, AF-related adverse events commonly result in acute medical conditions requiring ED visits or hospitalizations. The healthcare burden from AF-related hospitalizations and its temporal trends have been well described in previous studies [9,10,28]. However, data about ED visits in AF patients are limited, especially from Asia.

ED visits are a relevant indicator reflecting acute clinical situations due to AF-related adverse events or poorly controlled AF symptoms. To understand the actual burden of AF-related acute complications and establish appropriate management strategies to reduce these acute complications to improve quality of life of AF patients, more comprehensive data about ED visits of AF patients are needed. Although previous studies have described the trends of ED visits for AF, these studies have generally focused on the general epidemiology of ED visits for AF as a primary cause or primary diagnosis, and not ED visits in patients with AF from several acute adverse events [12,13,29]. Furthermore, most studies were performed in Western countries, and contemporary data from the Asian AF population are limited.

In this study, we have described the annual numbers of AF patients who visited the ED from any cause. The crude numbers of patients with ED visits has been rapidly growing with the increase in AF prevalence, consistent with previous studies [12,13,29]. Indeed, not only ED visits but also AF hospitalization rates significantly increased between 2006 and 2015 (relative increase, 468%) in Korea [9]. The increase in the number of ED visits might be attributable to the increasing total AF population, aging, and the increasing prevalence of comorbidities including hypertension, diabetes, heart failure, and CKD. Patients with ED visits showed high-risk profiles, including older age and a higher prevalence of comorbidities. Reflecting on their high-risk profile, the rate of OAC use was higher in patients with ED visits. One of the possible reasons for the higher OAC use rate in patients with ED visits compared to those who did not attend ED might be an increased recognition of AF-related complications during their regular healthcare contact. Thirty-five percent of patients who visited the ED ≥1 time had revisited the ED in the same year. From ED visits, subsequent hospitalizations occurred in 65% to 75% of total ED visits, and this was consistent with previous studies that 60% to 70% of ED visits resulted in hospital admission [12,13].

Stroke was the leading cause of ED visits in patients with AF. The proportion of patients with ED visits due to cerebral infarction as a primary diagnosis was 4% among total AF patients. Considering the mean CHA_2_DS_2_-VASc score of 3.8 for patients with ED visits and 3.4 for total AF population in 2017, 4% of stroke rate was consistent with previous reports [20,30]. The proportion of patients with a prior history of stroke in AF patients varied from 10% to 40% depending on the population, despite similar mean CHADS or CHA_2_DS_2_-VASc score [1,3,4,9,20].

Despite an increased proportion of high-risk patients (CHA_2_DS_2_-VASc score of ≥2 being 77.8% in 2006 to 86.3% in 2017), the 30-day and 90-day mortality were improved during the study period, which was consistent with previous reports [9,29,31]. This might be attributable, in part, to improvements in general medical care and the development of optimal management strategies for AF patients [32]. Especially, an increase in overall OAC use and NOAC prescription could be one of the major reasons to improve mortality after ED visits [31]. However, this should not be interpreted as a direct causality because improvements in general patient care other than OACs (e.g., increase of statin use) may also contribute to the better survival of AF patients with ED visits. Indeed, implementation of optimal risk assessment and management workflow improves clinical outcomes in patients with AF who have visited the ED [33,34,35]. However, these approaches are still not well validated from a large-scale prospective perspective. Also, we need further research to analyze the reason(s) for mortality reduction and improvement in clinical outcomes of AF patients with ED visits.

Although the annual incidence of ED visits was stable at 30%, the causes of ED visits have been changing over time. During the 12-year study period, ED visits for ischemic stroke, ICH, and MI decreased, whereas ED visits for GI bleeding and other major bleeding slightly increased but were generally stable. These temporal changes were consistent with previous reports [9,31] and seem to coincide with the introduction of NOAC. Over the last decade, overall OAC prescriptions have increased, and NOACs comprise the majority of OAC treatment [31,36]. Overall, the NOACs show comparable efficacy and better safety than warfarin, and the benefits are more accentuated in Asians than in non-Asians [21,37,38]. Along with these changes, thromboembolic complications such as ischemic stroke and MI decreased over time. Despite the increased OAC use, the risk of ICH decreased, and other bleeding events did not significantly increase, possibly related to the safety of NOAC.

Cardiovascular and AF-related adverse events were highly ranked among causes of ED visits, but non-cardiovascular causes such as pneumonia, COPD, or CKD, and trauma-related complications including fracture or intracranial injury, also commonly resulted in ED visits. A previous French study reported that non-cardiovascular deaths accounted for 43% of the causes of death for patients with AF [39]. Given the common causes of ED visits in AF patients, more holistic or integrated management approaches of underlying cardiovascular and non-cardiovascular comorbidities are warranted to improve patient care in AF.

### Study Limitations

This study had several limitations. First, AF and other comorbidities were defined by claims database using operational definitions based on ICD-10-CM codes. Therefore, the possibility of misclassification could not be excluded. To minimize possible errors, we used definitions validated and widely used in previous studies [3,4,5,9,15,19,21]. Nonetheless, miscoding and undercoding could occur in the study based on the claims database, and the results should be interpreted with caution. Although the accuracy of diagnostic codes was different in different settings, the accuracy for ICD-9 codes for stroke with concurrent AF diagnosis was 82%, stroke attributable to AF was 73%, and primary stroke codes were more accurate than nonprimary codes (97% versus 84%, *p* < 0.001) [40]. Second, 109 AF was identified using diagnostic codes. We did not include more detailed information about AF 110 status, such as type of AF (paroxysmal, persistent, or long persistent), AF duration, and AF burden 111 given the inherent limitations of the claims database. Third, we analyzed bleeding complications, including ICH, GI bleeding, and other major bleeding events as one of the AF-related complications. We assumed that it is not possible to differentiate those with treatment-related or spontaneous complications. Therefore, it would be appropriate to analyze all these events without considering the modality of treatment. Also, most of the AF patients who visited ED were prescribed antithrombotic therapy. Lastly, we did not identify the cause of death because of the inherent limitation of the study database. Despite these limitations, this study is strengthened by the use of a well-established Asian large-scale nationwide single-payer dataset to describe temporal trends of ED visits in patients with AF.

## 5. Conclusions

A substantial proportion of patients with AF attended ED every year, and the annual numbers of AF patients who visited the ED significantly increased over 12 years. With an increase of OAC utilization, the incidence of ED visits due to ischemic stroke, ICH, and MI have decreased. Including guideline-adherent OAC utilization and optimized management approaches in a holistic and integrated manner should be provided to reduce ED visits of AF patients.

## Figures and Tables

**Table 1:** Baseline characteristics of patients who visited ED.

	2006	2007	2008	2009	2010	2011	2012	2013	2014	2015	2016	2017	*p* for Trend
**Number**	40,425	44,576	47,975	52,648	57,754	59,914	67,011	70,376	79,188	87,077	99,306	99,763	<0.001
**Number of ED visits**													
**Mean** **± SD**	1.6 ± 1.1	1.6 ± 1.1	1.6 ± 1.1	1.6 ± 1.1	1.6 ± 1.1	1.6 ± 1.3	1.7 ± 1.6	1.7 ± 1.3	1.7 ± 1.2	1.7 ± 1.5	1.7 ± 1.6	1.6 ± 1.3	<0.001
**1**	66.5	65.9	65.4	65.0	64.2	63.4	63.1	63.3	63.3	63.1	62.0	66.3	<0.001
**2**	21.5	21.8	21.8	22.0	22.2	22.5	22.5	22.3	22.5	22.4	22.4	21.1	0.937
**3**	7.1	7.3	7.6	7.6	7.7	8.1	8.1	8.2	8.0	8.1	8.4	7.1	<0.001
**4**	2.7	2.8	2.8	3.0	3.2	3.2	3.3	3.2	3.3	3.3	3.6	2.8	<0.001
**≥5**	2.2	2.3	2.4	2.5	2.7	2.8	3.0	3.0	3.1	3.2	3.6	2.7	<0.001
**Age**													
**Mean** **± SD**	68 ± 14	68 ± 14	69 ± 14	69 ± 14	70 ± 13	70 ± 13	71 ± 13	71 ± 13	72 ± 13	72 ± 13	72 ± 13	73 ± 13	<0.001
**20–29**	1.5	1.4	1.4	1.2	1.0	0.9	0.8	0.7	0.7	0.7	0.6	0.5	<0.001
**30–39**	3.0	2.8	2.6	2.4	2.1	2.1	1.9	1.8	1.6	1.5	1.4	1.3	<0.001
**40–49**	7.1	6.9	6.3	5.9	5.4	4.9	4.4	4.2	4.1	4.0	3.8	3.5	<0.001
**50–59**	12.7	12.4	12.0	11.6	11.7	11.9	11.7	11.4	11.2	10.3	10.2	9.6	<0.001
**60–69**	24.5	23.6	22.5	21.5	20.7	19.9	19.2	18.6	18.3	18.5	18.6	18.0	<0.001
**70–79**	32.6	33.3	34.2	34.8	35.2	35.4	35.6	36.1	35.0	34.2	33.1	32.6	<0.001
**80+**	18.6	19.6	21.1	22.6	23.9	25.0	26.4	27.3	29.1	30.8	32.3	34.6	<0.001
**Sex (male)**	54.3	54.4	54.3	53.9	54.5	54.5	54.5	55.0	54.9	54.9	54.5	54.8	<0.001
**Comorbidities**													
**Hypertension**	79.6	79.7	80.5	80.3	80.6	78.4	81.7	83.5	83.0	83.4	82.7	83.4	<0.001
**Diabetes**	28.4	28.7	29.4	29.6	30.3	30.1	30.9	31.1	31.2	31.3	31.5	32.2	<0.001
**HF**	23.0	23.2	22.7	22.5	22.5	23.5	23.9	25.6	26.7	29.2	31.4	35.1	<0.001
**Stroke/TIA/TE**	19.2	19.5	20.8	21.8	22.4	22.1	22.9	23.6	22.9	23.1	22.8	23.0	<0.001
**MI**	7.1	6.8	6.8	6.6	6.3	5.5	4.7	4.7	4.5	4.9	5.2	5.5	<0.001
**PAD**	11.6	13.7	16.6	19.3	20.0	19.6	18.5	18.9	19.3	20.7	21.8	22.3	<0.001
**VHD (EHRA type 2)**	5.5	5.2	5.1	4.8	4.7	4.9	5.0	4.9	4.8	4.8	4.7	4.6	<0.001
**Cancer**	8.0	8.7	8.7	9.1	9.4	9.4	9.0	8.7	8.8	8.7	9.0	9.2	<0.001
**COPD**	23.1	24.1	24.4	24.2	24.2	24.2	24.3	24.0	23.0	23.4	23.3	23.3	<0.001
**CKD**	4.8	5.1	5.3	5.7	5.9	6.4	7.1	7.7	8.3	8.8	9.2	10.1	<0.001
**HD**	1.7	1.8	1.8	2.0	2.1	2.1	2.4	2.5	2.6	2.7	2.8	2.8	<0.001
**PD**	0.5	0.4	0.4	0.4	0.4	0.4	0.4	0.3	0.3	0.3	0.3	0.3	<0.001
**Previous PCI**	1.9	2.1	2.4	2.4	2.6	2.6	2.7	2.8	2.7	2.7	2.6	2.7	<0.001
**CHA_2_DS_2_-VASc score**													
**Mean** **± SD**	3.1 ± 1.6	3.2 ± 1.7	3.3 ± 1.7	3.3 ± 1.7	3.4 ± 1.7	3.4 ± 1.7	3.4 ± 1.7	3.5 ± 1.7	3.5 ± 1.7	3.6 ± 1.7	3.7 ± 1.7	3.8 ± 1.7	<0.001
**0, 1**	18.3	17.8	16.4	15.8	15.5	16.0	14.6	13.5	13.2	12.3	12.0	10.9	<0.001
**≥2**	81.7	82.2	83.6	84.2	84.5	84.0	85.4	86.6	86.9	87.7	88.0	89.2	
**Medication**													
**Antithrombotic therapy**													
**No therapy**	24.7	23.3	20.7	19.5	18.9	17.5	16.6	15.2	15.5	15.4	14.5	13.7	<0.001
**Antiplatelet agents**	45.8	45.6	47.2	47.5	47.3	46.1	45.3	43.3	42.1	36.6	31.5	26.8	<0.001
**Oral anticoagulants**	29.5	31.1	32.1	33.0	33.8	36.4	38.1	41.5	42.4	48.0	54.0	59.5	<0.001
**Warfarin**	29.5	31.1	32.1	33.0	33.8	36.2	37.8	36.6	38.2	22.4	13.3	9.9	<0.001
**NOAC**	0	0	0	0	0	0.2	0.3	4.9	4.2	25.6	40.7	49.6	<0.001
**Antiarrhythmic drugs**													
**Class Ic**	6.5	7.0	7.6	8.4	9.4	10.9	11.0	12.0	12.7	12.5	13.1	13.8	<0.001
**Class III**	13.8	13.9	15.1	16.0	16.3	17.0	17.1	18.0	19.1	19.3	19.5	21.0	<0.001
**Digoxin**	44.4	42.2	40.7	38.8	36.8	35.6	34.1	32.2	30.9	29.3	26.8	25.8	<0.001
**Beta-blocker**	41.8	42.5	43.3	43.4	44.3	46.2	46.7	46.9	46.6	47.1	48.0	48.8	<0.001
**Non-DHP CCB**	26.8	26.8	27.4	28.1	28.8	29.3	27.9	27.7	27.1	26.4	26.1	25.7	0.004
**DHP CCB**	33.9	31.3	28.6	24.3	21.3	18.5	16.5	14.9	13.7	12.3	11.5	10.5	<0.001
**ACE inhibitor**	42.4	44.3	46.9	49.7	51.1	51.6	52.2	53.0	52.2	51.8	51.7	51.6	<0.001
**ARB**	43.1	43.5	43.3	41.5	40.6	39.7	39.1	37.8	36.6	36.3	36.0	36.7	<0.001
**Diuretics**	69.6	68.5	67.7	66.8	66.3	64.8	64.1	63.3	62.1	62.0	61.5	62.7	<0.001
**Statin**	28.4	30.9	32.5	35.5	38.1	39.9	42.9	46.0	48.8	50.5	52.4	54.2	<0.001

Abbreviation: ACE, angiotensin-converting enzyme; ARB, angiotensin II receptor blocker; CCB, calcium-channel blocker; CKD, chronic kidney disease; COPD, chronic obstructive pulmonary disease; DAPT, dual antiplatelet therapy; DHP, dihydropyridine; ED, emergency department; EHRA, European Heart Rhythm Association; HD, hemodialysis; MI, myocardial infarction; NOAC, non-vitamin K antagonist oral anticoagulant; PAD, peripheral artery disease; PD, peritoneal dialysis; PCI, percutaneous coronary intervention; SD, standard deviation; TE, thromboembolism; TIA, transient ischemic attack; VHD, valvular heart disease.

## Supplementary Materials

The following are available online at https://www.mdpi.com/2077-0383/9/5/1485/s1, Table S1. Definitions of covariates and clinical outcomes, Table S2. Baseline characteristics of the total AF population in Korea, Table S3. The annual number of patients who visited the ED, number of total ED visits, and clinical outcome including subsequent hospitalization, 30-day, and 90-day mortality after ED visit, Table S4. Comparison of baseline characteristics between patients who visited or did not visit ED in 2017, Table S5. Number of patients who visited the ED from AF-related complications from 2006 to 2017.
