# Supplementary material for: Temporal Trends of Emergency Department Visits of Patients with Atrial Fibrillation: A Nationwide Population-Based Study
Source: J Clin Med. 2020 May 14;9(5):1485. doi: 10.3390/jcm9051485 (PMC7290994; doi:10.3390/jcm9051485)
Supplement: Supplementary file 1 [file jcm-09-01485-s001.pdf]

Supplementary Materials

Temporal trends of emergency department visits of patients with atrial fibrillation: a nationwide population-based study

Supplementary Tables

Table S1. Definitions of covariates and clinical outcomes

Table S2. Baseline characteristics of the total AF population in Korea

Table S3. The annual number of patients visited ED, number of total ED visits, and clinical outcome including subsequent hospitalization, 30-day, and 90-day mortality after ED visit

Table S4. Comparison of baseline characteristics between patients who visited or did not visit ED in 2017

Table S5. Number of patients who visited the ED from AF-related complications from 2006 to 2017

Table S1. Definitions of covariates and AF-related complications

Diagnosis	ICD-10-CM codes
AF	I48.0-I48.4, I48.9
Valvular atrial fibrillation (EHRA type 1 VHD)	I05.0, I05.2, I05.9, Z95.2-Z95.4 with AF diagnosis
Hypertension	I10-I13, I15; and minimum 1 prescription of anti-hypertensive drug (thiazide, loop diuretics, aldosterone antagonist, alpha-/beta-blocker, calcium-channel blocker, angiotensin-converting enzyme inhibitor, angiotensin II receptor blocker).
Diabetes mellitus	E11-E14; and minimum 1 prescription of anti-diabetic drugs (sulfonylureas, metformin, meglitinides, thiazolidinediones, dipeptidyl peptidase-4 inhibitors, α-glucosidase inhibitors, and insulin).
Dyslipidemia	E78
Heart Failure	I50
Stroke/TIA/TE	I63, I64, G458, G459, I74
Myocardial infarction	I21, I22
Peripheral artery disease	I70, I73
VHD (EHRA type 2)	I34.0-I34.2 (mitral valve disease), I35.0-I35.2, I06.0-I06.2 (aortic valve disease), I36.0-I36.2, I07.0-I07.2 (tricuspid valve disease) and I37.0-I37.2 (pulmonic valve disease
Cancer	C00-97 and RID code (V193)
Chronic obstructive pulmonary disease	J41-J44
Chronic kidney disease	N18, N19
Hemodialysis	Procedure code: O7011-O7020
Peritoneal dialysis	Procedure code: O7017, O7075
Percutaneous coronary intervention	Procedure code: M6561, M6562, M6563, M6564
CHA_2DS_2-VASc score	Heart failure (1 point), hypertension (1 point), age ≥75 years (2 points), diabetes mellitus (1 point), previous stroke/TIA/TE (2 points), vascular disease (prior myocardial infarction or peripheral artery disease, 1 point), age ≥65 years (1 point) and female sex (1 point)
**AF-related complications (as a primary diagnosis of ED visit)**	
Ischemic stroke	I63, I64
Atrial fibrillation	I48.0-I48.4, I48.9
Heart failure	I50
Myocardial infarction	I21, I22
Intracranial hemorrhage	I60-62

Gastrointestinal bleeding	I85, K22.1, K22.8, K25.0, K25.2, K25.4, K25.6, K26.0, K26.2, K26.4, K26.6, K27.0, K27.2, K27.4, K27.6, K28.0, K28.2, K28.4, K28.6, K29.0, K31.8, K55.2, K57.0, K57.1, K57.2, K57.3, K57.4, K57.5, K57.8, K57.9, K62.5, K66.1, K92.0, K92.1, K92.2
Other major bleeding	D62 (posthemorrhagic anemia), H05.2 (hemorrhage of orbit), H35.6 (retinal hemorrhage), H43.1 (vitreous hemorrhage), J94.2 (hemothorax), M25.0 (hemarthrosis), R04 (hemorrhage from respiratory passages; epistaxis, hemorrhage from throat, hemoptysis, or other sites/unspecified sites from respiratory passages)

Abbreviation: AF, atrial fibrillation; ED, emergency department; EHRA, European Heart Rhythm Association; RID, Rare Intractable Disease program; VHD, valvular heart disease.

Table S2. Baseline characteristics of the total AF population in Korea

	2006	2007	2008	2009	2010	2011	2012	2013	2014	2015	2016	2017	P for trend
**Number**	132,548	144,842	157,368	170,953	186,147	193,346	214,900	229,947	256,790	285,713	317,017	347,709	
**Age**													
**Mean ± SD**	66±13	66±13	67±13	67±13	68±13	68±13	69±13	69±13	70±13	70±13	70±12	71±12	<0.001
**20-29**	1.5	1.4	1.2	1.0	0.9	0.8	0.7	0.6	0.6	0.6	0.6	0.5	<0.001
**30-39**	3.1	2.9	2.6	2.4	2.2	2.0	1.8	1.6	1.5	1.5	1.3	1.2	<0.001
**40-49**	8.0	7.7	7.0	6.5	6.0	5.5	5.1	4.7	4.5	4.4	4.2	3.9	<0.001
**50-59**	15.6	15.2	15.0	14.7	14.7	14.9	14.6	14.2	13.7	13.1	12.6	12.1	<0.001
**60-69**	28.1	27.4	26.9	26.3	25.4	24.4	23.5	23.0	22.9	23.2	23.3	22.9	<0.001
**70-79**	30.3	31.2	32.1	32.8	33.3	34.1	35.0	35.6	35.1	34.2	33.7	33.6	<0.001
**80-**	13.5	14.4	15.3	16.4	17.6	18.2	19.3	20.4	21.7	23.0	24.4	25.9	<0.001
**Sex (male)**	54.5	54.9	55.2	55.3	55.9	56.2	56.6	57.1	57.4	57.4	57.3	57.5	<0.001
**Comorbidities**													
**Hypertension**	76.2	75.9	76.4	77.1	77.6	74.5	78.7	80.7	80.3	79.8	79.7	79.6	<0.001
**Diabetes mellitus**	21.6	22.0	22.6	23.3	23.8	24.0	24.6	25.2	25.5	25.7	26.2	26.8	<0.001
**Heart failure**	24.1	23.5	22.6	22.0	21.9	22.8	23.3	25.0	26.1	28.9	31.0	33.9	<0.001
**Stroke/TIA/TE**	17.2	17.7	18.3	19.2	19.8	19.6	20.2	21.1	20.9	21.0	21.2	21.1	<0.001
**Myocardial infarction**	5.5	5.2	5.1	5.0	4.7	4.2	3.6	3.6	3.6	3.8	4.0	4.2	<0.001
**Peripheral artery disease**	10.5	12.3	15.1	17.9	18.5	18.1	17.2	17.5	17.6	18.6	19.5	19.9	<0.001
**VHD (EHRA type 2)**	5.6	5.0	4.9	4.7	4.6	4.7	4.8	4.8	4.6	4.5	4.3	4.1	<0.001
**Cancer**	5.7	6.4	6.6	7.0	7.2	7.4	7.0	6.7	6.8	6.8	6.9	7.0	<0.001
**COPD**	19.5	20.2	20.2	20.0	19.9	19.8	19.8	19.7	18.5	19.0	18.7	18.8	<0.001
**Chronic kidney disease**	2.9	3.1	3.3	3.6	3.8	4.0	4.6	5.1	5.5	5.8	6.3	6.9	<0.001
**Hemodialysis**	0.9	0.9	1.0	1.0	1.1	1.1	1.3	1.3	1.4	1.4	1.5	1.6	<0.001
**Peritoneal dialysis**	0.2	0.2	0.2	0.2	0.2	0.2	0.2	0.2	0.1	0.2	0.2	0.1	<0.001
**Previous PCI**	1.5	1.7	1.9	2.1	2.0	2.0	2.1	2.2	2.2	2.2	2.1	2.1	<0.001
**CHA_2DS_2-VASc score**													
**Mean ± SD**	2.9±1.6	2.9±1.6	3±1.6	3±1.6	3.1±1.6	3.1±1.7	3.1±1.6	3.2±1.6	3.3±1.6	3.3±1.6	3.4±1.7	3.4±1.7	<0.001
**0, 1**	22.2	21.7	20.6	19.8	19.3	20.1	18.4	16.7	16.2	15.6	14.8	13.7	<0.001
**≥2**	77.8	78.3	79.4	80.2	80.7	79.9	81.6	83.3	83.8	84.4	85.2	86.3	
**Medication**													
**Antithrombotic therapy**													
**No therapy**	24.1	22.8	21.0	20.2	19.2	18.3	17.0	15.5	15.6	16.0	15.3	14.9	<0.001
**Antiplatelet agent**	46.8	47.4	49.0	49.3	49.6	48.7	48.6	47.2	46.3	40.6	35.0	30.1	<0.001
**Oral Anticoagulants**	29.1	29.8	30.0	30.5	31.2	33.0	34.4	37.3	38.1	43.4	49.7	55.0	<0.001

**Warfarin**	29.1	29.8	30.0	30.5	31.1	32.8	34.1	33.5	34.9	22.7	15.4	11.4	<0.001
**NOAC**	0	0	0	0	0.1	0.2	0.3	3.8	3.2	20.7	34.3	43.6	<0.001
**Antiarrhythmic drugs**													
**Class Ic**	7.2	7.6	8.3	8.9	9.9	11.3	12.0	12.9	13.5	13.7	14.4	15.2	<0.001
**Class III**	10.1	10.3	10.5	10.7	10.8	11.1	11.2	11.6	12.3	12.4	12.5	13.1	<0.001
**Digoxin**	41.2	38.6	36.5	34.6	32.4	31.2	29.5	27.5	25.8	23.8	21.7	20.0	<0.001
**Beta-blocker**	36.0	36.2	36.7	37.0	38.2	39.9	40.7	40.7	40.8	41.3	42.5	43.1	<0.001
**Non-DHP CCB**	19.2	19.2	19.6	19.8	20.1	20.2	19.7	19.4	18.7	18.2	17.9	17.2	<0.001
**DHP CCB**	26.4	24.2	21.9	18.7	16.4	14.1	12.6	11.5	10.5	9.5	8.7	7.9	<0.001
**ACE inhibitor**	38.7	41.0	43.5	46.5	48.1	49.0	49.8	50.7	49.9	49.4	49.8	49.9	<0.001
**ARB**	34.6	35.3	35.0	33.8	32.8	31.5	30.3	29.1	27.8	26.8	26.5	26.3	<0.001
**Diuretics**	58.5	56.4	54.8	53.4	52.2	50.5	49.3	48.5	47.1	46.3	46.2	45.9	<0.001
**Statin**	23.8	26.5	28.3	31.6	34.3	36.1	39.0	42.0	45.1	47.0	49.1	50.7	<0.001

Abbreviation: ACE, angiotensin-converting enzyme; ARB, angiotensin II receptor blocker; CCB, calcium-channel blocker; COPD, chronic obstructive pulmonary disease; DAPT, dual antiplatelet therapy; DHP, dihydropyridine; EHRA, European Heart Rhythm Association; NOAC, non-vitamin K antagonist oral anticoagulant; PCI, percutaneous coronary intervention; SD, standard deviation; TE, thromboembolism; TIA, transient ischemic attack; VHD, valvular heart disease.

Table S3. The annual number of patients visited ED, number of total ED visits, and clinical outcome including subsequent hospitalization, 30-day, and 90-day mortality after ED visit

	2006	2007	2008	2009	2010	2011	2012	2013	2014	2015	2016	2017
**Total AF patients**	132,548	144,842	157,368	170,953	186,147	193,346	214,900	229,947	256,790	285,713	317,017	347,709
**Number of patients who visited ED ≥1 in index year**	40,425	44,576	47,975	52,648	57,754	59,914	67,011	70,376	79,188	87,077	99,306	99,763
**% of patients with ≥1 ED visit in index year**	30.5%	30.8%	30.5%	30.8%	31.0%	31.0%	31.2%	30.6%	30.8%	30.5%	31.3%	28.7%
**Number of total ED visits of NVAF patients**	62,781	69,781	75,806	83,721	93,102	97,903	111,101	116,102	130,441	144,724	169,678	159,004
**Subsequent hospitalization after ED visit**	46,024	49,907	53,376	57,724	63,882	65,667	73,283	75,779	84,798	95,917	126,727	119,432
**% of subsequent hospitalization after ED visit in total ED visits**	73.3%	71.5%	70.4%	68.9%	68.6%	67.1%	66.0%	65.3%	65.0%	66.3%	74.7%	75.1%
**30-day mortality after ED visit**	10.4%	10.3%	10.0%	9.6%	9.8%	9.6%	9.6%	8.9%	8.8%	8.6%	8.2%	7.6%
**90-day mortality after ED visit**	16.3%	15.9%	15.5%	15.1%	15.6%	15.3%	15.1%	14.3%	14.0%	13.8%	13.2%	12.0%

Abbreviation: NVAF, non-valvular atrial fibrillation; ED, emergency department.

Table S4. Comparison of baseline characteristics between patients who visited or did not visit ED in 2017

	Total	Patients who did not visit ED	Patients who visited ED	p-value
**Number**	347,709	247,946	99,763	
**Age**				
**Mean ± SD**	71±12	70±12	73±13	<0.001
**20-29**	0.5	0.5	0.5	<0.001
**30-39**	1.2	1.2	1.3	
**40-49**	3.9	4.1	3.5	
**50-59**	12.1	13.0	9.6	
**60-69**	22.9	24.9	18.0	
**70-79**	33.6	34.0	32.6	
**80-**	25.9	22.4	34.6	
**Sex (male)**	57.5	58.6	54.8	<0.001
**Comorbidities**				
**Hypertension**	79.6	78.1	83.4	<0.001
**Diabetes mellitus**	26.8	24.6	32.2	<0.001
**Heart failure**	33.9	33.5	35.1	<0.001
**Stroke/TIA/TE**	21.1	20.3	23.0	<0.001
**Myocardial infarction**	4.2	3.7	5.5	<0.001
**Peripheral artery disease**	19.9	18.9	22.3	<0.001
**COPD**	18.8	17.0	23.3	<0.001
**Chronic kidney disease**	6.9	5.5	10.1	<0.001
**Hemodialysis**	1.6	1.1	2.8	<0.001
**Peritoneal dialysis**	0.1	0.1	0.3	<0.001
**Previous PCI**	2.1	1.8	2.7	<0.001
**CHA_2DS_2-VASc score**				
**Mean ± SD**	3.4±1.7	3.3±1.6	3.8±1.7	<0.001
**0, 1**	13.7	14.8	10.8	<0.001
**≥2**	86.3	85.2	89.2	
**Medication**				
**Antithrombotic therapy**				<0.001
**No therapy**	14.9	15.5	13.7	
**Antiplatelet agent**	30.1	31.3	26.9	
**Oral Anticoagulants**	55.0	53.2	59.5	
**Warfarin**	11.4	12.0	9.9	
**NOAC**	43.6	41.2	49.6	

**Antiarrhythmic drugs**				
**Class Ic**	15.2	15.7	13.8	<0.001
**Class III**	13.1	9.9	21.0	<0.001
**Digoxin**	20.0	17.6	25.8	<0.001
**Beta-blocker**	43.1	40.8	48.8	<0.001
**Non-DHP CCB**	17.2	13.8	25.7	<0.001
**DHP CCB**	7.9	6.8	10.5	<0.001
**ACE inhibitor**	49.9	49.2	51.6	<0.001
**ARB**	26.3	22.1	36.7	<0.001
**Diuretics**	45.9	39.2	62.7	<0.001
**Statin**	50.7	49.3	54.2	<0.001

Abbreviation: ACE, angiotensin-converting enzyme; ARB, angiotensin II receptor blocker; CCB, calcium-channel blocker; COPD, chronic obstructive pulmonary disease; DAPT, dual antiplatelet therapy; DHP, dihydropyridine; NOAC, non-vitamin K antagonist oral anticoagulant; PCI, percutaneous coronary intervention; SD, standard deviation; TE, thromboembolism; TIA, transient ischemic attack.

Table S5. Number and incidence of patients who visited the ED from AF-related complications from 2006 to 2017

	2006	2007	2008	2009	2010	2011	2012	2013	2014	2015	2016	2017
**Ischemic stroke**	5,393 (4.07)	5,829 (4.02)	6,440 (4.09)	6,667 (3.90)	7,513 (4.04)	7,812 (4.04)	8,661 (4.03)	9,006 (3.92)	9,746 (3.80)	10,167 (3.56)	11,395 (3.59)	10,911 (3.14)
**AF**	3,948 (2.98)	4,542 (3.14)	5,008 (3.18)	5,539 (3.24)	6,162 (3.31)	7,202 (3.72)	7,895 (3.67)	8,065 (3.51)	9,283 (3.62)	9,675 (3.39)	11,058 (3.49)	11,256 (3.24)
**HF**	2,412 (1.82)	2,693 (1.86)	2,960 (1.88)	3,224 (1.89)	3,671 (1.97)	3,734 (1.93)	4,063 (1.89)	4,315 (1.88)	4,804 (1.87)	5,340 (1.87)	6,310 (1.99)	6,216 (1.79)
**MI**	1,967 (1.48)	1,898 (1.31)	1,634 (1.04)	1,510 (0.88)	1,491 (0.80)	1,428 (0.74)	1,401 (0.65)	1,470 (0.64)	1,701 (0.66)	1,796 (0.63)	2,061 (0.65)	2,018 (0.58)
**ICH**	1,195 (0.90)	1,154 (0.80)	1,128 (0.72)	1,102 (0.64)	1,202 (0.65)	1,139 (0.59)	1,167 (0.54)	1,139 (0.50)	1,261 (0.49)	1,331 (0.47)	1,320 (0.42)	1,243 (0.36)
**GI bleeding**	1,056 (0.80)	1,164 (0.80)	1,235 (0.78)	1,362 (0.80)	1,530 (0.82)	1,529 (0.79)	1,750 (0.81)	1,853 (0.81)	2,109 (0.82)	2,296 (0.80)	2,670 (0.84)	2,438 (0.70)
**Other major bleeding**	399 (0.30)	516 (0.36)	527 (0.33)	612 (0.36)	623 (0.33)	687 (0.36)	861 (0.40)	859 (0.37)	1,032 (0.40)	1,130 (0.40)	1,263 (0.40)	1,197 (0.34)

Number (%)

The incidence was defined as ED visits from AF-related complications per 100 patients with AF (%).

Abbreviation: AF, atrial fibrillation; GI, gastrointestinal; HF, heart failure; ICH, intracranial hemorrhage; MI, myocardial infarction.
